# Cricothyrotomy Is Faster Than Tracheostomy for Emergency Front-of-Neck Airway Access in Dogs

**DOI:** 10.3389/fvets.2020.593687

**Published:** 2021-01-11

**Authors:** Sureiyan Hardjo, Catriona Croton, Solomon Woldeyohannes, Sarah Leonie Purcell, Mark David Haworth

**Affiliations:** ^1^School of Veterinary Science, The University of Queensland, Gatton, QLD, Australia; ^2^Faculty of Health, Engineering and Sciences, School of Sciences, University of Southern Queensland, Toowoomba, QLD, Australia

**Keywords:** cricothyrotomy, CICO, BACT, tracheostomy (TT), eFONA, airway obstruction, difficult airway, intubation

## Abstract

**Objectives:** In novice final year veterinary students, we sought to: (1) compare the procedure time between a novel cricothyrotomy (CTT) technique and an abbreviated tracheostomy (TT) technique in canine cadavers, (2) assess the success rate of each procedure, (3) assess the complication rate of each procedure via a damage score, (4) evaluate the technical difficulty of each procedure and (5) determine the preferred procedure of study participants for emergency front-of-neck access.

**Materials and Methods:** A prospective, cross-over, block randomised trial was performed, where veterinary students completed CTT and TT procedures on cadaver dogs. Eight students were recruited and performed 32 procedures on 16 dogs. A generalised estimating equation approach to modelling the procedure times was used.

**Results:** The procedure time was significantly faster for the CTT than the TT technique, on average (*p* < 0.001). The mean time taken to complete the CTT technique was 49.6 s (95% CI: 29.5–69.6) faster on average, with a mean CTT time of less than half that of the TT. When taking into account the attempt number, the procedure time for a CTT was 66.4 s (95% CI: 38.9–93.9) faster than TT for the first attempt, and for the second attempt, this was 32.7 s (95% CI: 15.2–50.2) faster, on average. The success rate for both procedures was 100% and there was no difference detected in the damage or difficulty scores (*P* = 0.13 and 0.08, respectively). Seven of eight participants preferred the CTT.

**Clinical Significance:** CTT warrants consideration as the primary option for emergency front-of-neck airway access for dogs.

## Introduction

An upper airway obstruction not responsive to conventional oxygen supplementation or airway management is termed a “cannot intubate, cannot oxygenate” (CICO) event (1). Surgical airway access, known as emergency front-of-neck airway access (eFONA) is indicated to provide oxygen as soon as possible (1). Such scenarios are rare in veterinary medicine and to the authors' knowledge, there are no current publications detailing clinical cases of eFONA in small animals. A review of the literature revealed multiple studies assessing tracheostomy (TT) procedures, however, all were performed on animals with a secured airway (2–6).

The TT is the current recommendation for eFONA in small animals (7, 8). Although cannula airway access is another method used to supply oxygen, clinical success in human emergencies is as low as 37% (9) and ventilation cannot be easily sustained (10). There is little mention of the surgical tube cricothyrotomy (CTT) in the veterinary literature, yet this is the current eFONA standard in the 2015 Difficult Airway Society (DAS) guidelines in human medicine (1). This procedure involves a surgical incision into the cricothyroid space and the insertion of an endotracheal tube. The CTT is rapid, easy to perform, and has minimal complications in human medicine due to the superficial anatomical landmarks targeted in the procedure (11–13).

A novel scalpel-bougie CTT technique has been proposed for veterinary use (14) however, it has not been directly compared to TT in the dog. A prospective, randomised cross-over experimental study assessing the performance of final-year veterinary students naïve to the TT and CTT procedures was conducted on cadaver dogs. The primary goal of the study was to assess the difference in procedure time between CTT and TT in dogs. The secondary outcomes were success rate, observation of damage, comparison of difficulty and procedure preference. The hypothesis was the CTT would be significantly faster but have a lower success rate than TT.

## Materials and Methods

Eight veterinary students were recruited and acted as their own controls in a cross-over fashion. Block randomisation was performed prior to participant recruitment using an online tool^1^ such that half performed CTT first and half performed TT first, with a block size of two and four chosen. Cadaver dogs were reused from completed wet labs.

Cadavers were paired targeting body weight within 5 kg of each other and body condition score within two points on a nine-point scale. A cadaver pair was allocated to a student, then a coin was flipped to assign the TT or CTT procedure to a cadaver. Each cadaver was placed in dorsal recumbency with a towel between the cervical spine and table. Instruments for each procedure were laid out and the ventral neck area of each cadaver was clipped. A fresh cadaver was used for the first attempt of either procedure. Second attempts utilised the same cadavers but crossed over the procedure (see Table 1). If a large incision or dissection was used in the first attempt, the skin was pulled across the midline and secured with towel clamps so that underlying anatomy was not visible for the second procedure.

**Table 1:** An example of cadaver usage during the study.

	**Cadaver 1**	**Cadaver 2**
Student 1 (first round)	CTT performed on fresh cadaver	TT performed on fresh cadaver
Student 1 (second round)	CTT incision present TT performed on used cadaver	TT incision present CTT performed on used cadaver

Materials provided for the CTT procedure included a 50 cm rigid 2.5 mm external diameter polypropylene dog urinary catheter, uncuffed endotracheal tube (ETT) cut to 7 cm in length, with the size dependent upon patient body weight (see Table 2) and a #10 scalpel blade on a #3 scalpel handle.

**Table 2:** ET tube sizes (internal diameter) based on body weight.

**Body weight (kg)**	**Tube size (mm)**
10–15	4.0
16–20	5.0
>20	6.0

Materials provided for the TT procedure included an ETT, #10 scalpel blade on a #3 scalpel handle, Mayo scissors, Gelpi retractors and Kelly haemostats. A minor surgical kit was within reach if the participant required additional instruments.

### Instruction

Students received a 10-min instructional oral presentation directly before each procedure, which contained information on basic regional anatomy, photos and videos. They performed the corresponding procedure immediately following these instructions. The presentation was not repeated for the second round. Copies of the presentations can be found in the Supplementary Materials.

Consistent with similar studies (11), students were encouraged to palpate surface landmarks on multiple animals before commencing the procedure. The students were specifically instructed to familiarise themselves with operating all equipment and to practise the hand motions of the technique to ensure equipment was not a limiting factor.

### Recording

Timing commenced from the first incision and ceased when the student verbally indicated completion with the word “stop.” The elapsed time was called at each 30 s interval by a researcher to add an element of urgency to the simulated emergency and any complications recorded. Success was classified as the tip of the endotracheal tube lying within the airway. The cadavers were examined by the researchers following the procedure who recorded damage scores and success of placement. A partial dissection was performed after the first round to visualise the tube in the airway and any luminal damage. Following both rounds, the entire cervical airway was opened on midline for examination to ensure damage was not overlooked.

Damage scores were adapted from a similar veterinary study (15). A score was given for each injury (Table 3). Students were given feedback on technique, damage and outcome for each procedure following examination by researchers.

**Table 3:** Post-procedural damage scoring system.

0	no gross damage
1	minor damage e.g., tracheal abrasions, scratches, off-midline incision, minor laceration of muscle <5 mm
2	moderate damage e.g., dorsal tracheal lesions, partial thickness mucosal laceration <5 mm, laceration of muscle >5 mm, deformation of tracheal rings
3	severe damage e.g., full-thickness tracheal tear, tracheal ring or cricoid cartilage fracture, oesophageal tear, incision between-tracheal rings greater than half the circumference of trachea

The students then completed a survey which included a visual analogue scale (VAS), scored from 0 to 10 with 0 being “the easiest procedure you can think of” and 10 being “the most technically challenging procedure you can imagine.” This method was adopted from previous similar studies (15, 16). Additionally, students were also asked which technique they would select in practice if faced with a CICO event.

### CTT and TT Technique

A modification to the CTT technique as described by Hardjo et al. (14) utilised the prominent caudal border of the cricoid cartilage as the key landmark for the laryngeal handshake. Identification of the larynx began by tracing the index finger of the non-dominant hand along midline of the ventral neck starting caudally and moving in a cranial direction until the cricoid cartilage was identified. The cricothyroid membrane (CTM) was then palpated as the small and soft depression immediately cranial to the cricoid cartilage and the procedure proceeded as previously described. A video link of the novel laryngeal handshake technique is provided in the footnotes.^2^

A brief description of the scalpel-bougie CTT technique used is as follows:

1. The CTM was located using the novel laryngeal handshake technique;
2. A #10 scalpel blade was used to make a 3–4 cm incision through the skin and soft tissues overlying the CTM;
3. A stab incision was made in to the CTM and a 50 cm polypropylene dog urinary catheter was immediately passed into the incision, and
4. An endotracheal tube was passed into the airway over the urinary catheter, similar to a modified Seldinger technique.

A video demonstrating this technique can be found in the Supplementary Materials. The TT technique was performed as the standard comparator and was adapted from several sources (8, 15, 17). As speed of placement was the primary objective, an abbreviated procedure was developed, colloquially known as a “slash” TT.

The slash TT prioritised exposure of the trachea with insertion of the tube in the fastest time possible. Exposure of the tracheal incision is a key step in performing a TT and is typically facilitated with suture placement around adjacent tracheal rings for retraction. In our abbreviated procedure, this was substituted by placing gelpi retractors at the level of the tracheal incision and haemostats were utilised to expose the stoma.

1. The larynx was palpated, being the first firm structure on the ventral midline neck moving cranially from the manubrium;
2. A ventral midline skin incision was made through the skin and subcutaneous tissues, beginning at the caudal larynx and extending 5–10 cm caudally (the participants were encouraged to make the incision as large as necessary to visualise the anatomy);
3. The sternohyoideus was incised on midline and the muscle was blunt-dissected with Mayo scissors to expose the trachea;
4. Gelpi self-retaining retractors were used to retract the soft tissues laterally at the level of the TT;
5. The membrane between the 3rd and 4th or 4th and 5th cartilage rings was incised, not exceeding 50% circumference;
6. Haemostats were used to depress the cranial aspect of the tracheal incision, and
7. The tube was inserted through the incision in a caudal direction.

The participants were informed that the use of haemostats to expand the tracheal incision was not essential if they judged the tube could be inserted more expediently without it.

### Statistical Analysis

A sample size calculation for the primary outcome resulted in the requirement for seven procedures in each group to achieve 80% power for detecting a difference in mean times of 60 s or less for CTT, and at least 120 s for TT. Sixty seconds was estimated from the author's experience and similar studies (18–20). The TT time estimate was based on the study by Pardo et al. (15), less the expected time saved with the abbreviated technique.

Analyses were conducted in R Statistical program software^3^ and Stata version 16.1^4^.

Data were summarised in accordance with their distribution and type, with normally distributed data as mean and standard deviation (SD), non-normal data as median and interquartile range (IQR) and where appropriate, categorical/binary data as proportion (%).

**Figure 1:**
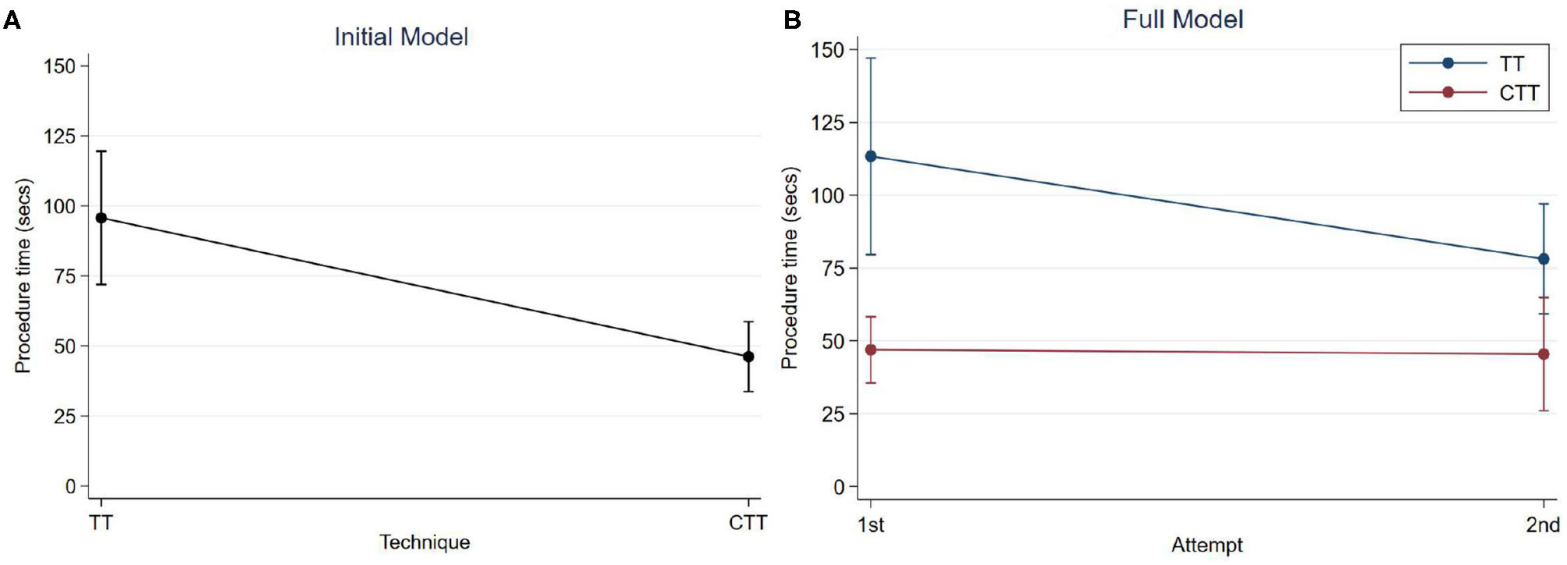
Predicted mean procedure time with 95% confidence intervals; **(A)** Initial model, by technique; **(B)** Full model, by technique and attempt.

To model the procedure times for CTT and TT for eFONA, a generalised estimating equation approach was used with the marginal regression model fitted using robust variances to protect validity of inferences in case of misspecification of the correlation structure. The procedure time for CTT and TT was the outcome variable, with the explanatory variable for the initial model being procedure type. For the full model, the explanatory variables added were attempt number (either first or second) and an interaction term between procedure and attempt number. Both models are shown in Table 4 and the marginal means in Figure 1. Residuals were examined for both models; there was no concerning deviation from normality. The non-parametric Mann Whitney U Test was performed to compare damage scores and the independent *t*-test was performed to compare difficulty scores between CTT and TT procedures. The significance level was set at 0.05.

**Table 4:** General estimating equation model estimates for initial and full models.

	**Initial model**			**Full model**		
	**Regression coefficient**	**95% CI**	***P* value**	**Regression coefficient**	**95% CI**	***P* value**
Constant (TT)	95.7	(71.9, 119.5)	<0.001	113.4	(79.6, 147.1)	<0.001
CTT	−49.6	(−69.6, −29.5)	<0.001	−66.4	(−93.9, −38.9)	<0.001
Attempt				−35.2	(−62.1, −8.4)	0.010
Interaction term				33.7	(10.8, 56.6)	0.004

## Results

Veterinary students consisted of six females and two males. There were seven students commencing final year of a five-year program and one graduand.

Sixteen canine cadavers were used, consisting of 15 entire females and one entire male. The gender imbalance was due to sourcing cadavers from ovariohysterectomy and female reproduction teaching. Breeds included nine Greyhounds, five mixed breeds, one Rottweiler and one American Staffordshire bull terrier. Body weights were normally distributed with a mean of 24.0 kg, SD of 3.93 and range of 15.2–32.2 kg. The cricothyroid spaces were also normally distributed with a mean of 13.1 mm, SD of 1.39 and range of 11.0–15.5 mm.

**Figure 2:**
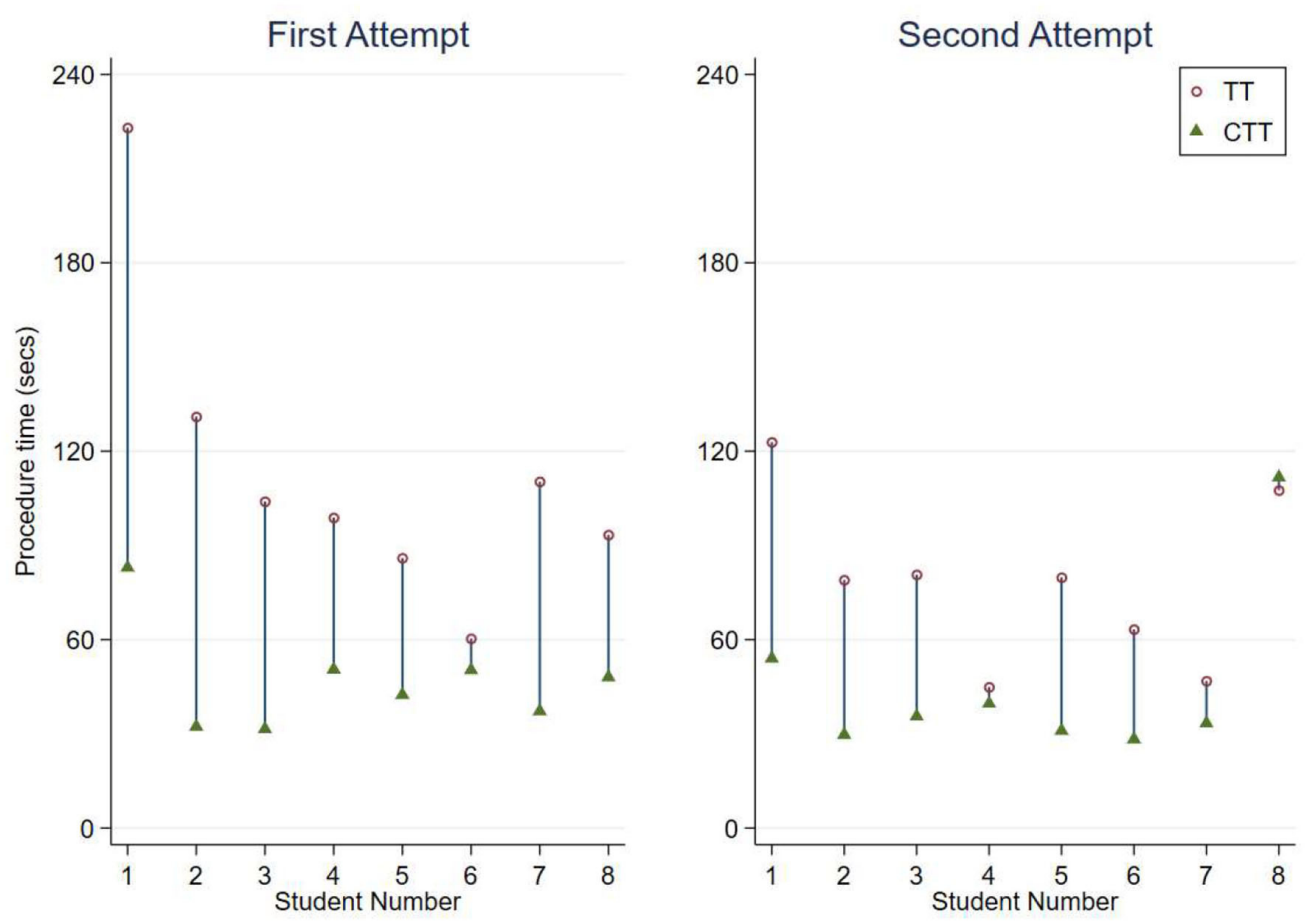
Pairs plot showing procedure times for each student, by attempt.

The procedure times are shown on Figure 2, categorised by first and second attempts. All procedures performed were successful.

The initial model showed very strong evidence of a difference in mean procedure times between techniques (*p* < 0.001). The procedure time for a CTT was 49.6 s (95% CI: 29.5–69.6) faster than for TT, on average (Table 4). The mean procedure time for a TT was 95.7 s (95% CI: 71.9–119.5) and for a CTT was 46.2 s (95% CI: 33.7–58.7), on average without considering attempt number (Figure 1A).

The additional variables of attempt number, and an interaction term between technique and attempt time were added to the initial model. This full model showed very strong evidence of a difference in mean procedure time between techniques for both the first and second attempts (*p* < 0.001 for both tests). For the first attempts, the procedure time for a CTT was 66.4 s (95% CI: 38.9–93.9) faster than for TT and for the second attempt this was 32.7 s (95% CI: 15.2–50.2) faster, on average. While the 95% confidence intervals of the mean time taken for the second attempts at TT and CTT overlap, the test of significance is for the difference between these means.

As shown in Figure 1B, the estimated mean time for the first attempt at a TT was 113.4 s (95% CI: 79.6–147.1), and for the second attempt was 78.1 s (95% CI: 59.3–97.0). The estimated mean time for the first attempt at a CTT was 46.9 s (95% CI: 35.6–58.3), and for the second time was 45.4 s (95% CI: 26.0–64.8).

When comparing completion times between TT attempts, there is strong evidence of a difference in mean procedure time for the second attempt compared to the first (*p* = 0.01). For the TT procedure, the time was 35.2 s faster (95% CI: 8.4–62.1) on average for the second attempt compared to the first. For the CTT attempts, the procedure time of the second attempt was an average of 1.5 s faster (95% CI: 18.2 slower to 21.2 faster) than the first and this difference was not significant (*p* = 0.9).

### Damage, Difficulty, and Procedure Preference

Damage was recorded in five of sixteen CTT attempts and nine of sixteen TT attempts. One student preferred the TT, stating the anatomy was more visible. Seven of eight preferred CTT, all stating it was the easier technique. There was no difference between damage and difficulty scores (Table 5).

**Table 5:** Damage and difficulty scores.

	**CTT**	**TT**	***P*-value**
Damage: Median (IQR)	0 (1)	1 (1.5)	0.13
Difficulty: Mean (SD)	2.6 (1.19)	3.8 (1.17)	0.08

**Figure 3:**
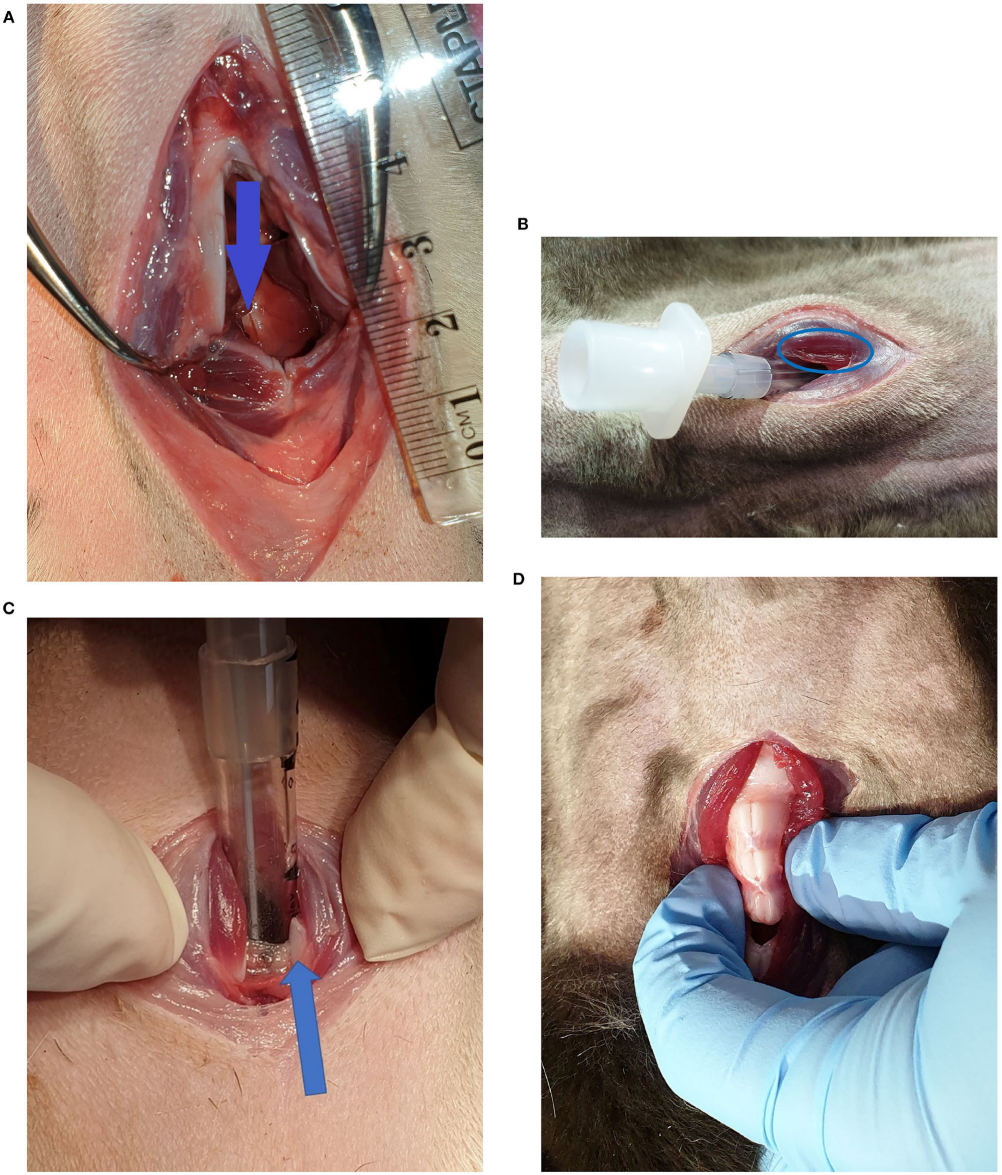
**(A)** Superficial injury on the luminal aspect of the dorsal cricoid cartilage, indicated by the arrow (CTT). **(B)** Sternohyoid muscle laceration (CTT). **(C)** Ventral cricoid transection (CTT). The arrow indicates one of the cut surfaces of the ventral cricoid cartilage. **(D)** Tracheal ring transections cranial and caudal to the tracheotomy incision (TT).

Some notable examples of damage are shown in Figure 3. There was one incident of intra-airway injury from a CTT shown in Figure 3A; a superficial mucosal laceration on the luminal aspect of the dorsal cricoid cartilage. Figure 3B shows a muscle laceration >5 mm sustained during CTT, graded a damage score of 2. Figure 3C depicts a ventral cricoid transection sustained during CTT, which was designated a damage score of 3. Multiple tracheal rings were transected in one of the TT procedures (Figure 3D), however the luminal mucosa remained intact. All other damage recorded was minor, consisting of muscle lacerations and superficial scratches on cartilage. There were 11 CTT procedures and 7 TT procedures with a damage score of 0.

## Discussion

The results of this study found that the bougie assisted surgical CTT using the method as described by Hardjo et al. (14) was significantly faster than a slash TT in canine cadavers on the first attempt at an airway surgery by veterinary students. Time to completion of the CTT was significantly faster than TT by an average of 49.6 s, which is less than half the time taken to perform the TT. Although there was a significant reduction in time between the first and second TT attempts, CTT procedures were still significantly faster for both attempts. There was no difference in success rate, damage or difficulty scores between the two procedures. The CTT technique was also the preferred procedure for seven of the eight participants. Therefore, it is likely the CTT will be consistently faster and have an equivalent success rate when compared to the TT in clinical practice.

Students performed CTT faster in 15 of 16 paired procedures. However, the improvement between attempts for each procedure was significant only for TT, indicating practise has a greater impact on achieving optimal results for this procedure. While there was no significant improvement in times between CTT attempts, the CTT was significantly faster than both attempts at TT, indicating that minimal instruction and practise is required to achieve optimal results. This makes CTT an ideal option for eFONA considering the infrequency of CICO and limited opportunities for veterinarians to practise airway procedures. Student 8 was the only student to have a slower CTT time compared with TT in any paired attempt. Following the attempt, student 8 noted that a towel clamp obstructed their palpation of landmarks during the procedure, which resulted in uncertainty and time lost before performing the stab incision in to the CTM.

One study (21) identified that human operator factors contributed to all serious events arising from emergency airway management. Hence, an easy procedure with fewer steps would reduce the cognitive demand and potentially improve outcome in a stressful situation. Although there was no significant difference in difficulty scores, seven of eight students preferred CTT over TT and used the word “easier” when describing the reasons for their choice. This preference is likely for the following reasons: the larynx is more superficial than the trachea, hence less dissection and fewer procedure steps are required; the larynx can also be stabilised externally so the incision in to the CTM can be safely performed blindly. Furthermore, the use of a bougie allows direct passage into the airway and is known to increase speed and accuracy of the CTT, enhancing the ease of the minimally invasive approach (14, 22). Therefore, the relative ease of CTT compared with the TT may be conducive to reducing serious complications following an emergency surgical intervention for a CICO event.

Students achieved a 100% success rate for both techniques following a brief training session. Interestingly, the rapidity of the CTT did not diminish its success and suggests the CTT technique used in this study is easy to learn and is on par or better than techniques used in experimental and clinical studies in humans (18, 23–26). We hypothesised that the success rate for CTT would be lower as the anatomy would not be directly visualised as opposed to the more extensive dissection and subsequent visualisation associated with TT. A possible explanation for the high success rate for both techniques was the large proportion of greyhound cadavers used. This breed typically has minimal subcutaneous tissue and readily allowed palpation of surface landmarks. Another explanation includes the laryngeal surface anatomy of dogs which is very prominent, particularly the caudal cricoid border, and is easily palpable compared with humans and other experimental models (14, 27). In addition, the mean cricothyroid space in our cadavers was 13.1 mm, which is larger than the average of 10.1 mm found in adult male humans (28). These findings further conflict with a 2002 study (29), where human medical personnel had a relatively poor success rate of 69.8% in performing CTT in canine cadavers. The results suggest species-specific training and veterinary students' familiarity with dog anatomy may be the cause for this discrepancy.

While speed of tube placement is a priority, safety of the technique employed is also important. The lacerated tracheal rings observed during one of the TT procedures (Figure 3B) would likely have the most clinical impact of all damage observed during the study. It could potentially allow airway collapse if negative pressure were applied during spontaneous inspiration. The CTT that resulted in ventral cricoid transection (Figure 3C) was deemed a success as the tip of the tube was within the airway. However, despite the high damage score, transection of the ventral cricoid cartilage has no long-term clinical effect in dogs (30). Various muscle lacerations, such as those in Figure 3B, were parallel to the muscle fibre direction and were all unlikely to have a long-term clinical impact to the patient. There was only one incidence of an internal airway injury where there was a minor laceration in the mucosa overlying the luminal aspect of the dorsal cricoid (Figure 3A). This would not be considered a clinically significant injury. Because cadavers were used, the immediate effect of haemorrhage could not be assessed, however live animal studies indicate it is of little concern when performing CTT (14, 31). There was no damage detected for the second attempts of CTT and there were only superficial scratches noted over the tracheal rings for the second TT attempts.

A limitation in study design may include all procedures being performed in one session. This could have contributed to the significant improvement between attempts for the TT procedure. Days or weeks separating these attempts may have tempered this effect. However, increasing the time between repetition of these procedures would likely strengthen the conclusion CTT is significantly faster than TT and requires less practise due to the steeper learning curve for TT. Another limitation is the potential for bias in this study as the authors have previously proposed CTT as the new standard in eFONA over the TT in small animals. It is possible the authors may have somehow influenced the TT procedure which resulted in longer times. However, a similar study, also using veterinary students as participants (15), found that the standard TT procedure had a median time of 188 s following 10 training attempts on mannikins and one training attempt on a cadaver. The abbreviated TT in this study took a median time of 95.7 s over all attempts, indicating that this was taught in a way to maximise speed. Another limitation was the power calculation used was for the primary aim of detecting a difference in times between the two procedures. Therefore, the study may have been underpowered to detect a difference in success rate, damage or difficulty scores.

The use of final year veterinary students as a study population is a limitation as the results may not be generalisable to the wider population of veterinarians who may perform eFONA. Although it is acknowledged external validity may be reduced, the sample of veterinary students at the same stage of their education and naive to performing airway surgery improved internal validity of the study by increasing sample homogeneity. In practice, criticalists or soft tissue surgeons already proficient in TT may achieve airway access in adequate times when compared with applying a novel technique. More work must be done to generalise the results to a larger population of veterinarians with varying experience and ascertain whether CTT has a true time advantage over TT.

## Conclusions

When performed by novice veterinary students, the CTT using the Hardjo technique (14) was faster, had the same success rate and was the preferred procedure when directly compared with an abbreviated TT in canine cadavers. When a CICO is declared following multiple failed intubation attempts, eFONA must be considered to minimise the effect of prolonged tissue hypoxia. Any additional procedure time adds to the likelihood of cerebral hypoxia and cardiac arrest. A steeper learning curve for TT than CTT was demonstrated in this study and indicates that regular practise and/or greater surgical experience is more important in attaining an optimal time for TT. This is not always possible, and performance is likely to degrade without practise over time (27). Therefore, a procedure that is rapid at the outset is desirable. Given the minimal training required to achieve a more immediate proficiency coupled with the rapidity of CTT demonstrated in this study, the authors recommend CTT be considered as a viable alternative to TT for eFONA, especially for veterinarians with limited surgical experience or those not already proficient in performing TTs.

## Data Availability Statement

The raw data supporting the conclusions of this article will be made available by the authors, without undue reservation.

## Ethics Statement

This study was reviewed and approved by The University of Queensland Institutional Human Research Ethics Committee. The participants provided their written informed consent to participate in this study. The animal component was reviewed and approved by The University of Queensland Animal Ethics Committee; Production and Companion Animals (PCA).

## Author Contributions

SH, CC, and MH: conception and design of the study and manuscript preparation. SH, MH, and SP: execution of the experiment and data recording. SH, MH, SP, and CC: manuscript drafting and review. SW and CC: results and statistical analysis. All authors: contributed to the article and approved the submitted version.

## Conflict of Interest

The authors declare that the research was conducted in the absence of any commercial or financial relationships that could be construed as a potential conflict of interest.

## Footnotes

^1^Sealed Envelope Ltd. *Create a Blocked Randomisation List* (2019). Available online at: https://www.sealedenvelope.com/simple-randomiser/v1/lists.

^2^https://www.youtube.com/watch?v=IzDh4WSjmNU

^3^R Core Team. R*: A Language and Environment for Statistical Computin*g. Vienna, Austria: R Foundation for Statistical Computing Version 3.6.1 (2019).

^4^StataCorp. *Stata Statistical Software: Release 16*. College Station, TX: StataCorp LLC (2019).

## References

[1] Frerk C Mitchell VS McNarry AF Mendonca C Bhagrath R Patel A Difficult Airway Society 2015 guidelines for management of unanticipated difficult intubation in adults. Br J Anaesth. (2015) 115:827–48. 10.1093/bja/aev371 26556848 PMC4650961

[2] Nicholson I Baines S. Complications associated with temporary tracheostomy tubes in 42 dogs (1998 to 2007). J Small Anim Pract. (2012) 53:108–14. 10.1111/j.1748-5827.2011.01167.x 22283793

[3] Bird FG Vallefuoco R Dupré G Brissot H. A modified temporary tracheostomy in dogs: outcome and complications in 21 dogs (2012 to 2017. J Small Anim Pract. (2018) 59:769–76. 10.1111/jsap.12928 30184262

[4] Vieitez V Martín-Cuervo M López-Ramis V Ezquerra LJ. Acute upper airway obstruction due to retropharyngeal hematoma in a dog with Anaplasma species: a case study. BMC Vet Res. (2015) 11:257. 10.1186/s12917-015-0574-7 26452479 PMC4600258

[5] Guenther-Yenke CL Rozanski EA. Tracheostomy in cats: 23 cases (1998–2006). J Feline Med Surg. (2007) 9:451–7. 10.1016/j.jfms.2007.06.002 17693112 PMC10911508

[6] De Gennaro C Vettorato E Corletto F. Severe upper airway obstruction following bilateral ventral bulla osteotomy in a cat. Can Vet J. (2017) 58:1313–6. 29203943 PMC5680743

[7] Fudge M Chapter 197 - Endotracheal intubation and tracheostomy. In: Silverstein DC, Hopper K, editors. Small Animal Critical Care Medicine. 2nd ed St. Louis, MO: W.B. Saunders (2015). p. 1024–8.

[8] Mann FAT Flanders MM Temporary tracheostomy. In: Creedon JMB, Davis H, editors. Advanced Monitoring and Procedures for Small Animal Emergency and Critical Care. Chichester: John Wiley & Sons (2012). p. 306–17.

[9] Cook TM Woodall N Frerk C Major complications of airway management in the UK: results of the fourth national audit project of the Royal College of Anaesthetists and the Difficult Airway Society. Part 1: anaesthesia. Br J Anaesth. (2011) 106:617–31. 10.1093/bja/aer058 21447488

[10] Coté JC Eavey DR Todres DI Jones ED Cricothyroid membrane puncture: oxygenation and ventilation in a dog model using an intravenous catheter. Crit Care Med. (1988) 16:615–9. 10.1097/00003246-198806000-00011 3371027

[11] Heymans SF Feigl MG Graber MS Courvoisier MD Weber MK Dulguerov MP. Emergency cricothyrotomy performed by surgical airway–naive medical personnel: a randomised crossover study in cadavers comparing three commonly used techniques. Anesthesiology. (2016) 125:295–303. 10.1097/ALN.0000000000001196 27275669

[12] Nugent WL Rhee KJ Wisner DH. Can nurses perform surgical cricothyrotomy with acceptable success and complication rates? Ann Emerg Med. (1991) 20:367–70. 10.1016/S0196-0644(05)81656-7 2003663

[13] Brofeldt BT Panacek EA Richards JR. An easy cricothyrotomy approach: the rapid four-step technique. Acad Emerg Med. (1996) 3:1060–3. 10.1111/j.1553-2712.1996.tb03355.x 8922017

[14] Hardjo S Croton C Haworth MD. A pilot study evaluating the utility of a novel tube cricothyrotomy technique in providing ventilation in small animals using a live porcine model. Vet Med. (2019) 10:111–21. 10.2147/VMRR.S216551 31934552 PMC6711556

[15] Pardo MA Sumner JP Friello A Fletcher DJ Goggs R. Assessment of the percutaneous dilatational tracheostomy technique in experimental manikins and canine cadavers. J Vet Emerg Crit Care. (2019) 29:484–94. 10.1111/vec.12869 31259471

[16] Poole O Vargo M Zhang J Hung O. A comparison of three techniques for cricothyrotomy on a manikin. Can J Respir Ther. (2017) 53:29. 30996627 PMC6422212

[17] Fossum TW Surgery of the upper respiratory system. In: Fossum TW, editor. Small Animal Surgery. 2. 5th ed Philadelphia, PA: Mosby (2018). p. 833–83.

[18] Pairaudeau CF Mendonca C Hillermann C Qazi I Baker PA Hodgson RE. Effect of palpable vs. impalpable cricothyroid membranes in a simulated emergency front-of-neck access scenario. Anaesthesia. (2018) 73:579–86. 10.1111/anae.14218 29349776

[19] Añez Simón C Serrano Gonzalvo V Carrillo Luna LH Farré Nebot V Holgado Pascual CM. Results of a surgical cricothyrotomy workshop with a pig trachea model. Rev Esp Anestesiol Reanim. (2019) 66:129–36. 10.1016/j.redar.2018.09.003 30514575

[20] Hill C Reardon R Joing S Falvey D Miner J Cricothyrotomy technique using gum elastic bougie is faster than standard technique: a study of emergency medicine residents and medical students in an animal lab. Acad Emerg Med. (2010) 17:666–9. 10.1111/j.1553-2712.2010.00753.x 20491685

[21] Flin R Fioratou E Frerk C Trotter C Cook TM. Human factors in the development of complications of airway management: preliminary evaluation of an interview tool. Anaesthesia. (2013) 68:817–25. 10.1111/anae.12253 23682749

[22] Layng E Berrios M Kadish J Pester JM 102 STABCric 2: surgical technique against bougie cricothyrotomy. Ann Emerg Med. (2015) 66:S36-S. 10.1016/j.annemergmed.2015.07.134

[23] Chang SS Tong QJ Beh ZY Quek KH Ang BH. A bench study comparing between scalpel-bougie technique and cannula-to-Melker technique in emergency cricothyroidotomy in a porcine model. Korean J Anesthesiol. (2018) 71:289–95. 10.4097/kja.d.18.00025 29843506 PMC6078881

[24] Schober P Biesheuvel T de Leeuw MA Loer SA Schwarte LA. Prehospital cricothyrotomies in a helicopter emergency medical service: analysis of 19,382 dispatches. BMC Emerg Med. (2019) 19:12. 10.1186/s12873-019-0230-9 30674276 PMC6343329

[25] Lockey D Crewdson K Weaver A Davies G. Observational study of the success rates of intubation and failed intubation airway rescue techniques in 7256 attempted intubations of trauma patients by pre-hospital physicians. Br J Anaesth. (2014) 113:220–5. 10.1093/bja/aeu227 25038154

[26] Paix BR Griggs WM. Emergency surgical cricothyroidotomy: 24 successful cases leading to a simple ‘scalpel–finger–tube' method. Emerg Med Australas. (2012) 24:23–30. 10.1111/j.1742-6723.2011.01510.x 22313556

[27] Palmer L. Concepts of prehospital advanced airway management in the operational K9: a focus on cricothyrotomy. J Spec Oper Med. (2019) 19:99–106. 30859536 10.55460/KV13-RV6C

[28] Tayama N Kaga K Chan RW Titze IR. Geometric characterization of the laryngeal cartilage framework for the purpose of biomechanical modeling. Ann Otol Rhinol Laryngol. (2001) 110:1154–61. 10.1177/000348940111001213 11768707

[29] McCarthy MC Ranzinger M Nolan D Lambert CS Castillo M. Accuracy of cricothyroidotomy performed in canine and human cadaver models during surgical skills training. J Am Coll Surg. (2002) 195:627–9. 10.1016/S1072-7515(02)01337-6 12437248

[30] Romita MC Colvin SB Boyd AD. Cricothyroidotomy - its healing and complications. Surg Forum. (1977) 28:174–5. 617408

[31] Bjoraker GD Kumar BN Brown CDA. Evaluation of an emergency cricothyrotomy instrument. Crit Care Med. (1987) 15:157–60. 10.1097/00003246-198702000-00016 3802860

