# Supplementary material for: Cricothyrotomy Is Faster Than Tracheostomy for Emergency Front-of-Neck Airway Access in Dogs
Source: Front Vet Sci. 2021 Jan 11;7:593687. doi: 10.3389/fvets.2020.593687 (PMC7829300; doi:10.3389/fvets.2020.593687)
Supplement: Supplementary file 2 [file Data_Sheet_1.PDF]

Tube cricothyrotomy instructional

1

Background

- Emergency airway access is required when a patient cannot be intubated. E.g. during upper airway obstruction
- Approaching the airway surgically is required
- CTT is a new technique in veterinary medicine, which has not yet been widely accepted as the first choice for emergency airway access

2

- All equipment will be provided ready-to-use

3

- You will be timed for this procedure.
- The researcher will call out at each 30s interval

4

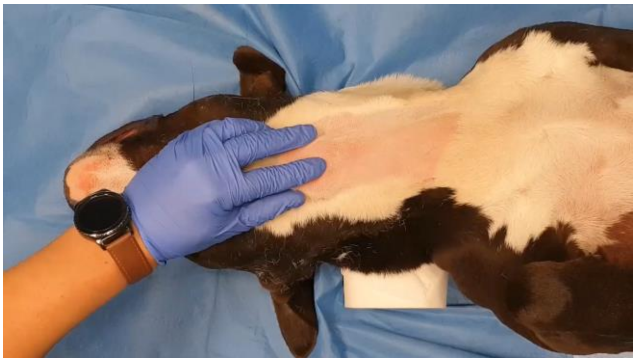

5

Anatomy

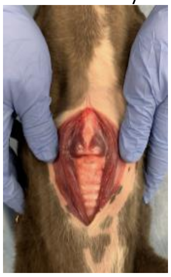

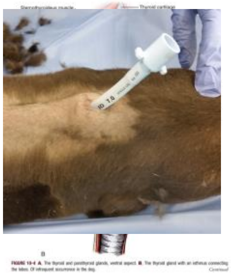

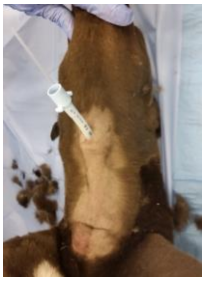

Wide approach (if you cannot feel structures)

Standard approach (mostly done by feel)

6

Summary

- Feel
- Slice
- Stab
- Slide

7

Feel

- RIGHT side of animal for right handers
- Left hand: Run index finger cranially from ventral neck
- First firm ring will be cricoid cartilage

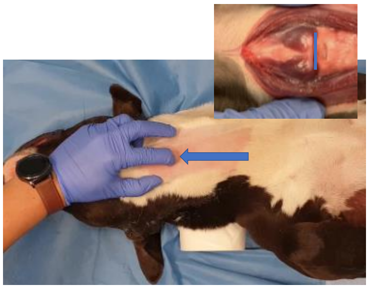

8

- Dip between cricoid and next firm structure cranially = cricothyroid notch

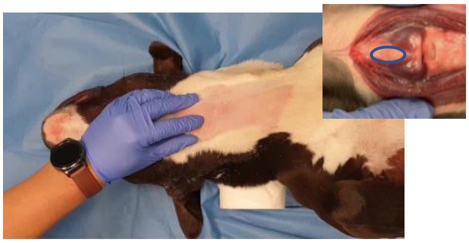

9

Incising

- Stabilize larynx by grabbing thyroid cartilage on either side
- 3-4cm incision
- Skin AND muscle
- Allows passage of the tube

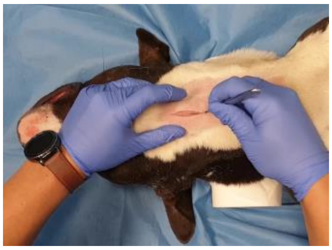

10

Incising through the membrane

- Feel for the notch again
- Stab and <u>leave the scalpel in situ</u>
- Needs to be deep enough
  - Feel a pop
  - Blade below the skin
- Arc or push caudally until resistance
- Cut needs to be long enough
  - If you feel cricoid straight away, rotate scalpel 180 degrees towards the head and incise 5mm

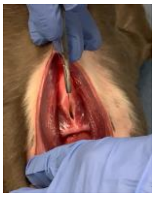

Dissected view - This is what it should look like below the skin

11

If you're unsure…

- You can keep dissecting until you see the larynx and membrane
- Or check by putting your finger entirely in to the airway through the incision

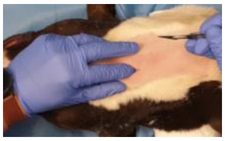

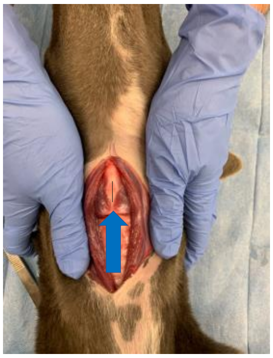

12

Pass U cath

- Push away from you with the flat side of scalpel
- U cath goes caudally into the incision. INSERT FOR 10cm

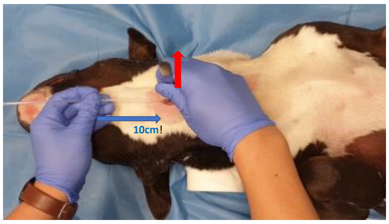

13

HOLD U CATH with non dominant hand
Slide in tube

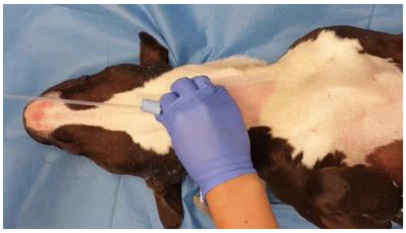

14

If it doesn't pass smoothly…

- Take it out and feel for the membrane again – place finger in the airway
- Stab and incise again

OR

- Bigger incision and dissection to see the larynx

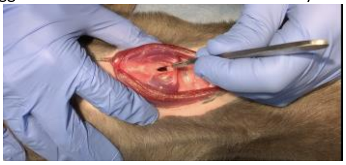

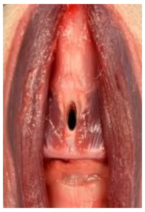

15

Indicate when you're done

- Say STOP!
- We will accept "I'm finished, stop timing, done". BUT please say STOP!

16

There are scores for damage

- Off midline incision
- Muscle transection (sideways cut rather than along fibres)
- Mucosal lacerations (within the airway)
- Damage to surrounding soft tissue structures

17

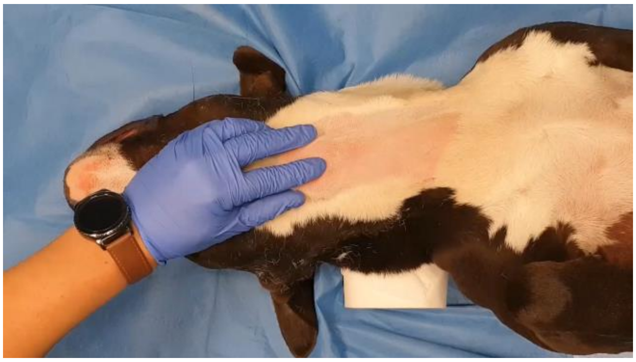

18
