# Supplementary material for: Cricothyrotomy Is Faster Than Tracheostomy for Emergency Front-of-Neck Airway Access in Dogs
Source: Front Vet Sci. 2021 Jan 11;7:593687. doi: 10.3389/fvets.2020.593687 (PMC7829300; doi:10.3389/fvets.2020.593687)
Supplement: Supplementary file 3 [file Data_Sheet_2.PDF]

Emergency Tracheostomy

1

Background

- Emergency airway access is required when a patient cannot be intubated. E.g. during upper airway obstruction
- Approaching the airway surgically is required
- TT is the recommended technique of choice in a life-threatening upper airway obstruction

2

- You will be timed for this procedure.
- The researcher will call out at each 30s interval

3

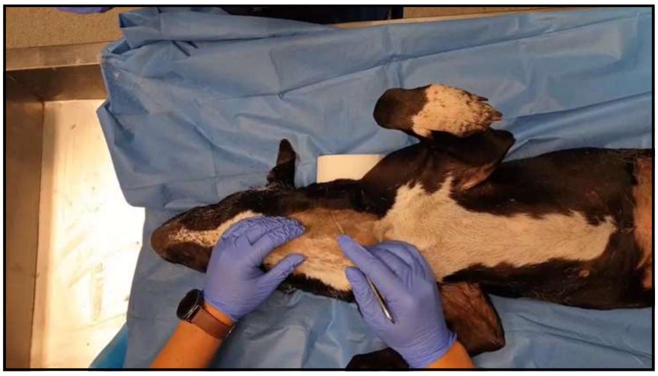

4

Anatomy

- This slide contained copyrighted material

5

Summary

- 5-10cm midline skin incision
- Incise between muscle
- Split with blunt dissection to expose trachea
- Retract with Gelpi
- Incise between the 4th and 5th tracheal rings
  - Do not exceed 50% circumference
- Insert tube

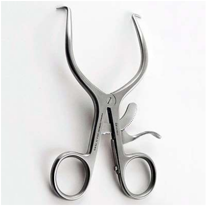

6

Landmarks

- Use larynx as the cranial landmark
- Difficult to feel trachea itself
- The incision should start 1cm caudal to this and continue 5-10 cm caudally
- Red box is the larynx
- Blue line is the incision

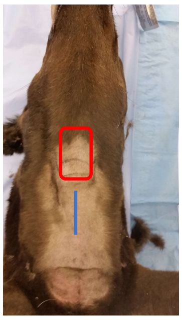

7

Incision begins 1cm caudally from larynx. 5-10 cm in length

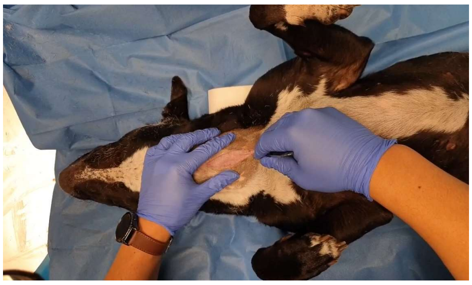

8

Try to split muscle between muscle fibres

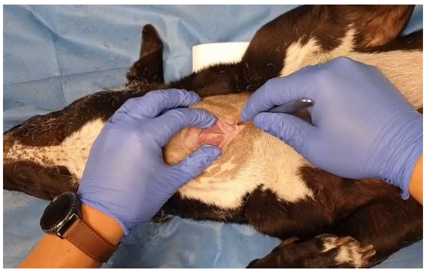

9

Blunt dissect and cut with scissors to expose trachea

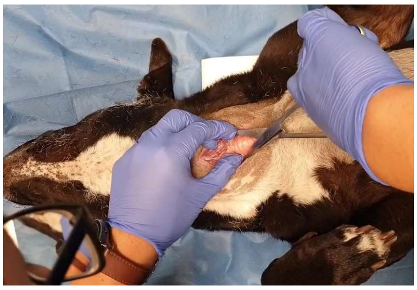

10

Retract with Gelpis at the level of tracheostomy

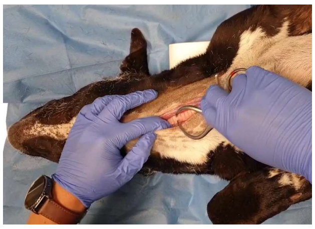

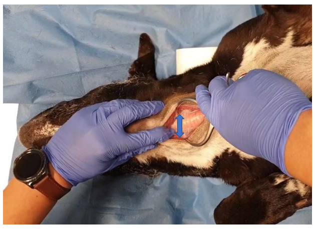

11

Tracheal incision between 3-4th or 4-5th rings

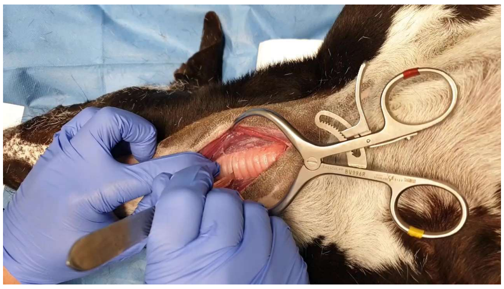

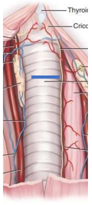

Location of tracheal incision

12

Pass tube

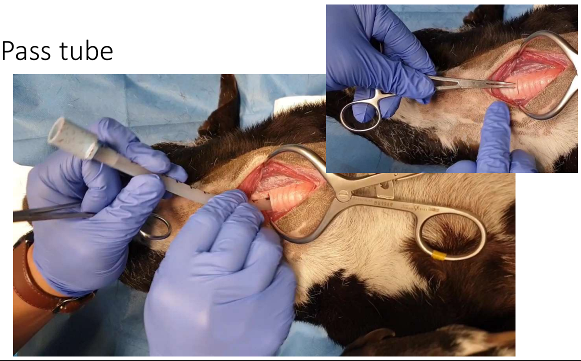

13

Indicate when you're done

- Say STOP!
- We will accept "I'm finished, stop timing, done". BUT please say STOP!

14

There are scores for damage

- Off midline incision
- Muscle transection (sideways cut rather than along fibres)
- Mucosal lacerations (within the airway)
- Damage to surrounding soft tissue structures

15

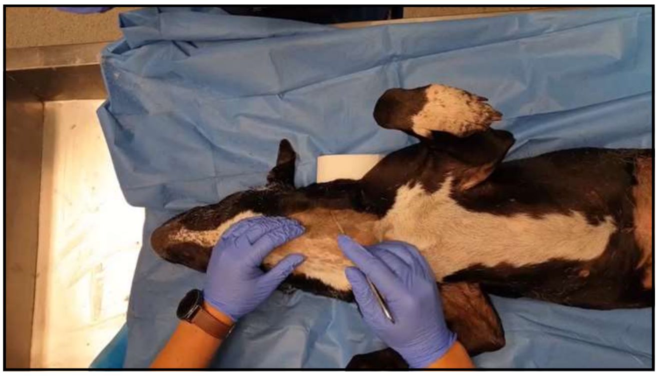

16

17
